# HDAC-Linked “Proliferative” miRNA Expression Pattern in Pancreatic Neuroendocrine Tumors

**DOI:** 10.3390/ijms19092781

**Published:** 2018-09-15

**Authors:** Eckhard Klieser, Romana Urbas, Stefan Swierczynski, Stefan Stättner, Florian Primavesi, Tarkan Jäger, Christian Mayr, Tobias Kiesslich, Pietro Di Fazio, Katharina Helm, Daniel Neureiter

**Affiliations:** 1 Institute of Pathology, Cancer Cluster Salzburg, Paracelsus Medical University/Salzburger Landeskliniken (PMU/SALK), Muellner Hauptstrasse 48, 5020 Salzburg, Austria; e.klieser@salk.at (E.K.); r.urbas@salk.at (R.U.); katharina.helm@pmu.ac.at (K.H.); 2 Department of Surgery, Paracelsus Medical University/Salzburger Landeskliniken (PMU/SALK), Muellner Hauptstrasse 48, 5020 Salzburg, Austria; stefan.swierczynski@gmail.com (S.S.); ta.jaeger@salk.at (T.J.); 3 Department of Visceral-, Transplant- and Thoracic Surgery, Medical University of Innsbruck, 6020 Innsbruck, Austria; stefan.staettner@tirol-kliniken.at (S.S.); florian.primavesi@tirol-kliniken.at (F.P.); 4 Department of Internal Medicine I, Paracelsus Medical University/Salzburger Landeskliniken (PMU/SALK), Muellner Hauptstrasse 48, 5020 Salzburg, Austria; christian.mayr@pmu.ac.at (C.M.); t.kiesslich@salk.at (T.K.); 5 Laboratory for Tumor Biology and Experimental Therapies, Institute of Physiology and Pathophysiology, Paracelsus Medical University (PMU), Strubergasse 22, 5020 Salzburg, Austria; 6 Department of Visceral, Thoracic and Vascular Surgery, Philipps University of Marburg, Baldingerstrasse 35043 Marburg, Germany; difazio@med.uni-marburg.de

**Keywords:** epigenetics, miRNA, histone deacetylases, pancreatic neuroendocrine tumor

## Abstract

Epigenetic factors are essentially involved in carcinogenesis, tumor promotion, and chemoresistance. Two epigenetic key players are miRNAs and histone deacetylases (HDACs). As previously shown by own theoretical databank analysis, the crosstalk between miRNAs and HDACs is relevant in different human chronic diseases and cancerogenic pathways. We aimed to investigate a potential connection between the expression of a well-defined subset of “proliferation-associated” miRNAs and the expression of HDACs as well as clinical parameters in pancreatic neuroendocrine tumors (pNETs). Materials and Methods: Expression levels of miRNA132-3p, miRNA145-5p, miRNA183-5p, miRNA34a-5p, and miRNA449a in 57 pNETs resected between 1997 and 2015 were measured and linked to the immunohistochemical expression pattern of members of the four HDAC classes on human tissue microarrays. All pNET cases were clinically and pathologically characterized according to published guidelines. Correlation analysis revealed a significant association between expression of specific miRNAs and two members of the HDAC family (HDAC3 and HDAC4). Additionally, a linkage between miRNA expression and clinico-pathological parameters like grading, TNM-staging, and hormone activity was found. Moreover, overall and disease-free survival is statistically correlated with the expression of the investigated miRNAs. Overall, we demonstrated that specific miRNAs could be linked to HDAC expression in pNETs. Especially miRNA449a (associated with HDAC3/4) seems to play an important role in pNET proliferation and could be a potential prognostic factor for poor survival. These first data could help, to improve our knowledge of the complex interactions of the epigenetic drivers in pNETs for further therapeutic approaches.

## 1. Introduction

Pancreatic neuroendocrine tumors (pNETs) are a rare group of neoplasms originating from the pancreas that exhibits endocrine phenotypes. They can be divided into functioning and non-functioning pNETs, with the latter representing the more common group. Functioning pNETs are characterized by specific hormonal hyper secretion and can be classified in insulinomas (45%), gastrinomas (20%), glucagonomas (13%), vasoactive intestinal peptide (VIP)-omas (10%), and somatostatinomas (5%) [1,2,3]. Incidence rates currently lie at 1–4 per 100,000 population per year but are increasing exponentially, mostly due to improved imaging techniques and concurrent detection rate [4]. Surgical resection is the primary therapeutic option for patients with pNET. Even though there are alternative and multidisciplinary therapeutic approaches, they have failed to provide prolonged survival benefit so far [5]. Due to this and the fact that surgery is not always applicable, there is an urgent need for new therapeutic targets and treatment options.

Aberrant epigenetic regulation of gene function is strongly linked to cancer initiation and progression [6,7]. Epigenetic malfunctions have been found to cause altered gene expression and epithelial to mesenchymal transmission (EMT). Epigenetic regulations can be mediated by DNA methylation, histone modifications or microRNAs (miRNAs) [8,9]. Especially, aberrations in histone modifying mechanisms such as histone de-/acetylation seem to play a significant role in cancer [10]. In the context of pNETs, histone deacetylation (and consequently silencing) of tumor suppressor genes may contribute to the formation of tumors arising from the exocrine and endocrine pancreas [11]. These deacetylation processes are regulated by a group of enzymes called histone deacetylases (HDACs), which can be allocated to four classes: Class I, IIA, IIB, III, and IV. Overexpression of HDACs has been reported in several cancer types including pNETs, making these enzymes potential therapeutic targets [12]. Another group of epigenetic regulating factors involved in cancerogenesis are miRNAs, which can function as both oncogenes and tumor suppressor genes (TSGs) [13,14,15,16,17,18]. MicroRNAs are single-stranded small non-coding RNAs with 18–25 nucleotides in length. Beyond their involvement in a diverse range of biological processes, miRNAs (oncogenic) are linked to several pro-tumorigenic processes, such as apoptosis resistance, cell proliferation, chemoresistance and EMT, dependent on the function of their respective target mRNA. Interestingly, previous studies demonstrated that some miRNAs directly interact with HDAC-mediated mechanisms by modulating genes encoding for particular HDACs [19,20]. Further research regarding these epigenetic mechanisms could lead to the development of improved treatment strategies by a combinatory approach targeting HDACs and specific miRNAs involved in cancer development and progression [21]. Based on these promising findings by other working groups regarding expression of specific miRNAs and HDAC expression, and based on the urgent requirement for new therapeutic strategies and/or targets in pNETs, we, therefore, aimed to investigate the expression levels of a well-defined subset of “proliferation-associated” miRNAs (miRNA132-3p, miRNA145-5p, miRNA183-5p, miRNA34a-5p, and miRNA449a) and their correlation with immunohistochemical expression patterns of members of the four HDAC classes. Moreover, we analyzed the association between these expression patterns and clinico-pathological parameters in pNETs.

## 2. Results

### 2.1. Clinical Characteristics of pNET Patients

As summarized in Table 1, 57 cases (*n* = 32 (56.1%) male and *n* = 25 (43.9%) female with a mean age of 60.9 ± 14.4 years) with pNET (*n* = 27 (47.4%) immunohistochemically endocrine positive) were included in the study. According to the WHO the pNETs were graded as NET G1 (*n* = 32 (56.1%)), NET G2 (*n* = 17 (29.8%)), and NEC (neuroendocrine carcinoma) G3 (*n* = 8 (14.0%)) with a mean Ki-67 associated proliferative and H&E/pHH3-associated mitotic activity of 10.8% and 12.8/15.0 per 10 high power fields. Related to the TNM, the pNET tumors had a mean size of 2.69 ± 2.34 cm, mainly located in the head of the pancreas (*n* = 25 (44.6%)) and presented mainly in TNM-stage T1 in *n* = 24 (42.1%) with a locoregional lymph node and distant metastasis rate in *n* = 19 (33.3%) and in *n* = 11 cases (19.3%), respectively. Finally, significant differences were observed for TNM, R status and events of overall survival (OS) and disease-free survival (DFS) inside the classical clinical parameters (using *χ*^2^ test, * *p* < 0.05).

### 2.2. Expression of miRNA in pNETs

**Figure 1:**
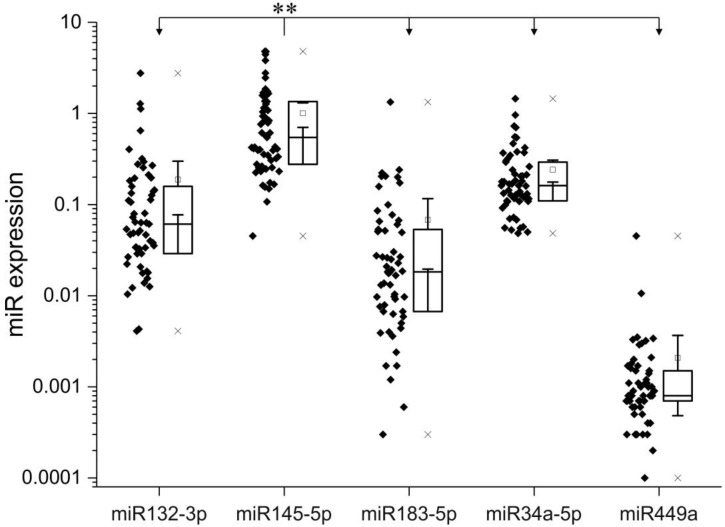
Evaluated expression of miRNA 132-3p, 145-5p, 183-5p, 34a-5p, 449a in the pancreatic neuroendocrine tumors (pNET) patient collective. Box plots showing the expression of individual miRNAs related to housekeeping genes. ** *p* < 0.01 (ANOVA, Bonferroni).

As shown in Figure 1, the miRNA expression analysis of the pNET samples with RT-PCR revealed that miRNA145-5p had the overall highest (significant difference, *p* < 0.001, ANOVA, Bonferroni) and miRNA449a, the overall lowest expression levels, respectively (expression levels in descending order: 145-5p, 34a-5p, 132-3p, 183-5p, 449a). For further analysis, the expression levels for each individual miRNA were separated into “low” and “high”, according to the mean expression score. The Youden-index was applied for calculating a significant cut-off-value for each miRNA, based on the OS and DFS rate (as listed in Table 2).

### 2.3. Correlation Analysis of miRNA and HDAC Expression

The correlation analysis between miRNA expression and HDAC expression patterns revealed that miRNA449a is significantly positively correlated with relative expression of HDAC3 (nuc) (+*(0.310)) and HDAC4(cyt) (+*(0.302)). Representative images of H&E morphology and HDAC3/4 immunohistochemistry in correlation with and miRNA449a stainings are shown in Supplementary Figure S2. Interestingly, we observed (by trend) higher expression levels of the remaining HDACs in our pNET samples (Supplementary Table S2).

### 2.4. Association of miRNA Expression with Grading and Mitotic/Proliferative Status

**Figure 2:**
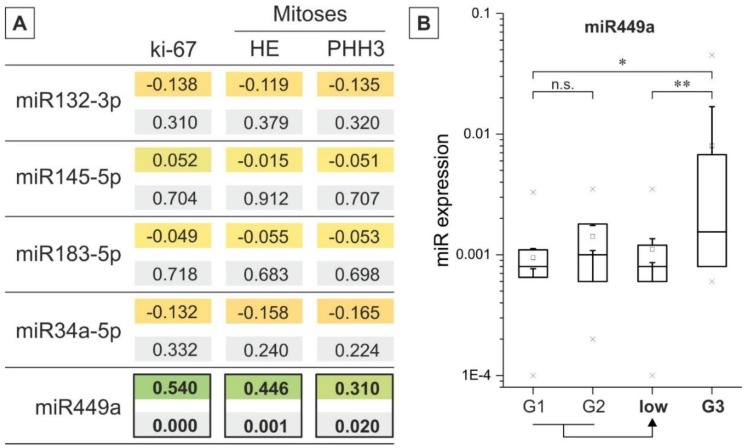
(**A**) Correlation analysis microRNAs (miRNA) expression and markers of proliferation and mitosis. The upper value stands for the Pearson correlation coefficient (absolute values), and the lower value is the related *p* value; significant differences are highlighted (black rectangles); (**B**) Quantitative analysis of pNET-miRNA expression versus grading. miRNA expression is plotted via box plots versus grading of pNET cases. Additionally, the expression levels were summarized for G1 and G2 pNET cases. * *p* < 0.05, ** *p* < 0.01 (*t*-Test and ANOVA).

All pNET tumor samples were classified according to the TNM-associated WHO-grading system (G1–G3), and their different miRNA expression levels were compared (see Figure 2 and Supplementary Figure S1). Furthermore, low-grade (G1 and G2) cases were grouped, and their miRNA expression level was compared to high-grade (G3). As illustrated in Figure 2, significant differences for miRNA449a between low- and high-graded tumors were observed, whereas remaining miRNAs showed heterogeneous expression patterns (Supplementary Figure S1). The miRNA145-5p, miRNA183-5p, and miRNA449a were upregulated in high-grade compared to low-grade pNETs, whereas miRNA132-3p was downregulated in high-graded pNETs compared to low-graded tumors. Of note, the expression of miRNA34a-5p remains stable in pNETs independent of the tumor grading. Interestingly, correlation analysis of miRNA expression and H&E associated mitotic and Ki-67 associated proliferative activity revealed (highly) significant associations for miRNA449a (Figure 2A): H&E mitoses (absolute +**(0.446)/relative +**(0.366)), PHH3 (absolute +*(0.310)/relative +*(0.283)), Ki-67 proliferative activity (absolute +**(0.540)/relative +*(0.330)).

### 2.5. miRNA Expression and Clinicopathological Characteristics

**Figure 3:**
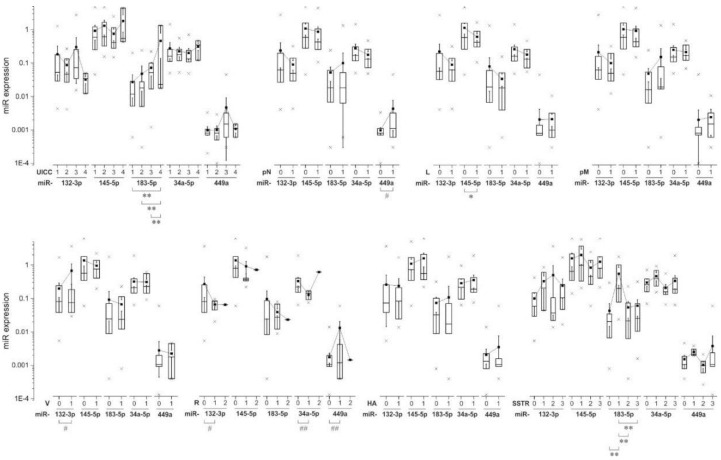
Relationship between miRNA expression and clinico-pathological data in pNET patients. Differences between expressions were tested using *t*-test or ANOVA and Bonferroni respectively. * *p* < 0.05, ** *p* < 0.01—equal variances assumed, # *p* < 0.05, ## *p* < 0.01—according to Levene variances not equal.

Next, we compared the absolute and relative miRNA expression with respect to clinico-pathological characteristics. If applicable, the relative miRNA expression levels were classified into low and high according to the mean expression score. Exemplary results are shown in Figure 3. We found that the expression of miRNA183-5p was highly significantly associated with the tumor size (low/high, +**(0.433), Pearson, data not shown). The Union International contre le cancer (UICC) status (categorized in 1–4) is correlated with higher miRNA183-5p-expression in an increasing manner (highly significant differences between groups 1-4 **(0.000), 2-4 **(0.001), 3-4 **(0.002); ANOVA, Bonferroni). In addition, we found a significant group difference between ENET status 1 and 4 (*(0.040); ANOVA, Bonferroni) and the expression of miRNA183-5p (data not shown). No significant differences were found regarding T-status (UICC and ENET) categorized into low and high (data not shown). Lymph node infiltration (pN) is linked to higher expression of miRNA449a (equal variances assumed *(0.047), no homogeneity of variances (Levene) (0.171); *t*-test). Invasion of lymphatic vessels (L) is significantly linked to lower expression of miRNA145-5p (*(0.024), *t*-test) but there are no significant differences related to podoplanin levels (data not shown). Analysis of the expression of our miRNAs in relation to distant metastasis (pM) did not reveal any significant differences. However, we found that pNET cases with vascular invasion (V) tend to have higher expression levels of miRNA-132-3p (*(0.045) no homogeneity of variances (Levene) (0.460)). The cell surface antigen CD34 was significantly coupled with increased expression levels of miRNA132-3p (*(0.019), *t*-test, data not shown). Regarding the resection margins status R0-R1 (there was only one pNET case with macroscopic residual tumor (R2). Therefore, a *t*-test was calculated between the groups R0 and R1) group differences were found for the expression levels of miRNA132-3p, 34-5p and 449a (*(0.017), **(0.000), **(0.002); no homogeneity of variances (Levene) (0.440, 0.186, 0.382); *t*-test, data not shown). pNET cases with hormone activity are by trend linked with higher expression of all miRNAs except miRNA132-3p. We then performed a correlation analysis between miRNA expression and the secretion of the hormones Somatostatin, Glucagon, Insulin, Serotonin, Gastrin, Calcitonin, and VIP. Somatostatin secretion was positively correlated with the expression of miRNA132-3p (relative −*(0.266)) and miRNA34a-5p (relative −*(0.272)). Levels of serotonin were positively correlated with miRNA145-5p- (absolute +**(0.409), relative +*(0.269)), miRNA183-5p- (absolute +**(0.944), relative +**(0.389)), miRNA34a-5p- (relative +*(0.304)) levels. Moreover, we found a positive correlation between Gastrin secretion and expression of miRNA34a-5p (+**(0.375)) (data not shown). The expression of the Somatostatin-receptor type 2 (SSTR2) can be categorized into four groups based on the expression levels (0, 1, 2, 3). According to ANOVA and Bonferroni analysis, there are highly significant differences regarding miRNA183-5p and SSTR2 expression (0-1 **(0.001), 1-2 **(0.002), 1-3 **(0.000)). Interestingly, SSTR2 status 1 with low expression levels is linked to the highest expression of miRNA183-5p.

### 2.6. Impact of miRNA Expression on DFS and OS

**Figure 4:**
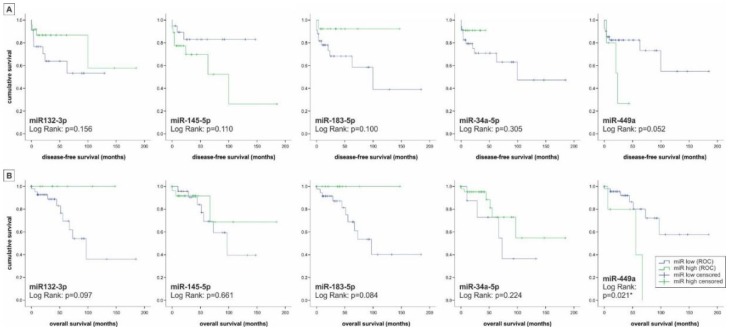
Survival analysis of patients with pNET. According to the classification of miRNAs in low or high based on the relative miRNA expression levels using receiver operating characteristic (ROC) calculation with Youden Index analysis the Kaplan–Meier survival analysis using the log–rank test was performed for (**A**) disease-free survival and (**B**) overall survival. “Protective miRNAs” include miRNA145-5p and miRNA183-5p, whereas “risk miRNAs” include miRNA132-3p, miRNA34a-5p, and miRNA449a. * *p* < 0.05, ** *p* < 0.01.

The OS and DFS status—in this part of the analysis standing for the number of deceased patients (0-1)—was significantly linked to a higher expression of miRNA449a (equal variances assumed *(0.012)/*(0.047) no homogeneity of variances (Levene) (0.272, 0.258); *t*-test). To evaluate the miRNAs 132-3p, 145-5p, 183-5p, 34a-5p, and 449a as potential prognostic markers for DFS and OS we performed Kaplan–Meier survival analysis based on the relative miRNA expression levels using receiver operating characteristic (ROC) calculation with Youden Index analysis (Figure 4). We found a significant association between the expression of miRNA449a and OS (0.036, log-rank (Mantel–Cox)). Interestingly, evaluation of miRNA expression and DFS and OS revealed two groups: Expression of miRNAs 132-3p, 183-5p and 34a-5p was associated with higher OS and DFS values (“tumor suppressor miRNAs”), whereas the expression level of miRNA449a was associated with reduced survival rates (“oncogenic miRNA”). Surprisingly the expression of miRNA145-5p was associated with lower OS and enhanced DFS.

Finally, time-dependent multivariate analyses were applied to identify statistically significant predictors for OS and DFS including miRNA and HDAC scoring as well as clinico-pathological parameters (grading, TNM-staging, resection status, tumor size, hormone activity, localization). In the first step, the Cox-regression analysis of OS including all these mentioned parameters indicated only the N-status (pN) (hazard ratio (HR): 692.3, 95% confidence interval (CI): 0.103–4657763) as the strongest and highly significant predictor for OS (*p* < 0.05, log-rank (Mantel–Cox) test). Including only miRNAs, miRNA449a (HR: 4.6, 95% CI: 1.1–19.6) alone and in binding with HDAC4 (cytoplasmatic) (HR: 1.02, 95% CI: 1.005–1.035) could be filtered out as a significant predictor for OS (*p* < 0.05, log-rank (Mantel-Cox) test). In the next step, the Cox-regression analysis of DFS showed again only the N-status (hazard ratio (HR): 52.6, 95% confidence interval (CI): 6.2–444.6) as the significant predictor for DFS in pNET-cases of all parameters (*p* < 0.05, log-rank (Mantel-Cox) test). Interestingly, no miRNA could be detected as a significant prognostic parameter for DFS, whereby only the binding of HDAC4 (cytoplasmatic) and miRNA499a (HR: 1.011, 95% CI: 0.999–1.024) reached significant levels (*p* < 0.05, log-rank (Mantel-Cox) test).

## 3. Discussion

pNET is a relatively rare tumor entity with increasing incidence rates. Current therapeutic routes are limited, underlining the need for new therapeutic targets and treatment options. In the present immunohistochemical study we present initial information about the potential role of five “proliferation-associated” miRNAs in pNETs and their link with specific tumor-related HDACs. Hereby, we not only provide first data about a general involvement of these miRNAs in development and progression of pNETs, but also connect these miRNAs to HDAC expression and thus to epigenetic (de-)regulation. This is especially interesting since in a previous study investigating HDAC-deregulation in pNETs, we found specific HDAC family members to be linked with tumor grading, patient survival, and proliferation markers [22]. The expression levels of the set of miRNAs within our cohort of pNET samples followed a descending order with miRNA145-5p being expressed the highest and miRNA449a, the lowest (expression levels in descending order: 145-5p, 34a-5p, 132-3p, 183-5p, 449a). Micro-RNA145-5p does have tumor suppressor function as analyzed in multiple types of cancer like bladder cancer, renal cell carcinoma, lung cancer, esophageal squamous cell carcinoma, and breast cancer, which is particularly interesting as it showed the highest expression levels in our samples [23,24,25,26,27,28]. In our cohort, the miRNA with the lowest expression levels—miRNA-449—is also very well cited in connection with multiple tumor entities except pNETs. According to several studies focusing on this miRNA, it has a role as a tumor suppressor and is downregulated in hepatocellular carcinoma, lung cancer, gastric cancer, prostate cancer and also pancreatic cancer [19,29,30,31,32]. Two studies found a correlation between miRNA449 down-regulation and HDAC1/HDAC3 up-regulation; two HDAC family members important for tumor cell proliferation, [19,29]. In our study the expression of miRNA449a is significantly positively correlated with HDAC3/HDAC4- expression. Of note, there are reports suggesting an oncogenic role also for HDAC4 (repression of the cell cycle inhibitor p21) [33]. Accordingly, the correlation between miRNA449a and HDAC3/4 seems to be of an up-regulating nature in our pNET cohort. Interestingly, miRNA449a is significantly positively correlated with mitotic and proliferative activity in our pNET sample cohort even though its main function in other tumor entities throughout the literature is tumor suppressive and cell growth inhibiting [34]. Our observation that miRNA449a expression is also correlated with lower survival rates and lymph node infiltration further suggests a pro-tumorigenic role of this particular miRNA in pNETs. When comparing low-grade (G1, G2) to high-grade (G3) pNET samples, we found that miRNA145-5p, miRNA183-5p, and miRNA449a were upregulated in high-grade compared to low-grade pNETs, whereby miRNA132-3p and miRNA34a-5p were downregulated in high-grade pNETs compared to low-grade pNETs. Micro-RNA132-3p is especially interesting as it harbors both, tumor-promoting as well as tumor-suppressing functions [35,36,37,38]. Regarding miRNA34a-5p, Bommer et al. demonstrated that this miRNA contributes to proper p53 function and that its inactivation is likely to be involved in cancer development, which is coherent with our findings in that miRNA34a-5p shows low expression in high-grade pNETs [39]. Our evaluations of OS and DFS in the pNET sample cohort revealed that besides miRNA449a, also miRNA183-5p, alone and interestingly, partially in binding with HDAC4 (for miRNA449a) is significantly associated with reduced OS and DFS in survival analysis with Cox Proportional-Hazards Model. This observation is interesting as not only miRNA449a could be established as a tumor suppressor in some cancer types but also miRNA183-5p, suggesting potential tumor-specific interactions and effects for these miRNA species. Studies analyzing the role of miRNA-183-5p in cancer have on the one hand revealed tumor suppressive functions, but on the other hand, miRNA183-5p seems to play a promoting role in EMT, cell migration and invasion [40,41]. In gallbladder cancer for example miRNA-183-5p is upregulated and involved in tumorigenesis according to a study by Gao et al. [42]. Similar findings were published regarding gastric cancer and esophageal squamous cell carcinoma [43,44,45]. However, there are also studies that describe miRNA183-5p as a tumor suppressor. For example, Yang et al. showed that miRNA-183 acts as a tumor suppressor in human non-small cell lung cancer [40]. Moreover, in an in vitro study with human pancreatic cancer cell lines, miRNA-183 showed an anti-EMT effect [46]. In our study, miRNA-183-5p expression is significantly upregulated in higher-grade pNET samples according to UICC standards but in contrast to that, it is also correlated with higher OS and DFS rates. Shi and coworkers investigated the expression of miRNA449 in breast cancer specimens and found its expression significantly associated with poor survival. In addition, they detected a pro-proliferative effect of miRNA449 on breast cancer cells in in vitro studies [47]. Similar observations were obtained by Wang et al: Treatment of MCF-7 breast cancer cells with a miRNA449a inhibitor led to a significantly reduction of cell proliferation and migration [48]. As there are no studies so far investigating the role of miRNA449a in pNETs and as there is evidence of other miRNAs not distinctly having either a tumor suppressive- or oncogenic function, the results of this study indicate, that miRNA449a does function as an oncogene in pNETs in an interplay with HDAC3 and HDAC4. To confirm this, underlying epigenetic mechanisms should be further investigated. Then miRNA449a could be established as a prognostic factor for poor survival in pNETs and it might even be an interesting potential therapeutic target in combination with HDAC3/4 inhibition.

## 4. Materials and Methods

### 4.1. Clinical and Pathological Characterization of pNET Cases

The cohort included *n* = 57 pNET cases that were surgically resected between 1997 and 2015 and archived at the Institute of Pathology, Paracelsus Medical University, Salzburg, Austria. All cases were comprehensively characterized for clinico-pathological parameters including sex, age, tumor size, localization, type of surgery, tumor grading, and staging. All cases were evaluated for grading (counting hematoxylin-eosin mitosis in 10 consecutive high power fields (HPF) according to published guidelines [49]), neuroendocrine differentiation as well as mitotic, proliferative and hormonal activity by immunohistochemistry on 5 μm formalin-fixed paraffin-embedded (FFPE) tissue sections as described in detail previously [50,51]. This study was conducted following the national and institutional guidelines of the Paracelsus Medical University Salzburg/Salzburg county hospital as well as in accordance with the declaration of Helsinki (1964); the anonymized samples are exclusively available for research purposes in retrospective studies. The analyses of human pNET samples were approved by the local ethics committee (415-EP/73/408-2014) (19 May 2014).

### 4.2. Isolation of Total miRNA from FFPE Samples and Expression Analysis by RT-PCR

Using a RM2245 semi-automated rotator microtome (Leica Biosystems Nussloch GmbH, Nussloch, Germany), one to three 10 μm sections (depending on tumor size) were cut from FFPE blocks of tumor samples after microdissection to remove surrounding tissue. These sections were transferred to 1.5 mL tubes, and subsequently, miRNA isolation was performed using the miRNeasy FFPE Kit. For subsequent RT-PCR analysis, microRNAs were transcribed utilizing the miScript II RT™ kit. All FFPE miR reagents and qPCR kits were obtained from Qiagen (Hilden, Germany) and used according to the manufacturer’s instructions.

### 4.3. Immunohistochemistry and Processing for Markers of HDACs (1-6, 8-11 and Sirt1)

To simplify the investigations and ensure comparability of the immunohistochemical signals, five tissue microarrays (TMA) covering all 57 cases were assembled. If possible, each pNET case was represented on the TMA with tumor tissue as well as adjacent healthy pancreatic tissue [52]. Each TMA was cut into 5 μm sections, raised on adhesive glass slides and dried at 60 °C for one hour. Deparaffinization, antigen retrieval, immunostaining, counterstaining, dehydration, and coverslip application, as well as pre-treatment, were performed using standardized routine immunohistochemistry (IHC) protocols. Immunohistochemical staining was performed on a Dako Autostainer Plus combined with the EnVision Plus System using primary mouse or rabbit antibodies (Dako, Vienna, Austria). In case of a primary goat antibody, a secondary enzyme conjugated antibody was used. Sources of all applied primary antibodies (including catalog-number, clone species, dilution, incubation, and pretreatment) are summarized in Supplementary Table S1.

### 4.4. Interpretation and Scoring of the IHC

Examination of each pNET case within the TMAs was carried out semi-quantitatively by counting the number of stained cells and assessing the intensity of the staining. According to the data sheets of the chosen HDAC antibodies, a cytoplasmatic (cyt: only HDAC 4, 5, 8, and 10) and/or a nuclear (nuc: all HDACs) expression pattern was separately evaluated. Afterwards an immunoreactivity score (range 0–300) was calculated by multiplying the scores for intensity (0–3) and the stained cells (extensity, 0–100%) as previously published [53]. In addition, absolute quantitative scoring data of each HDAC, the relative data of low and high HDAC expressions levels were estimated according to the expression’s means as HDAC-specific cut-off-values, as previously published [22].

### 4.5. Statistical Analysis

Statistical analysis was performed with SPSS 24.0 (IBM Corporation, New York, USA). The *χ*^2^-test (nominal), Student’s *t*-test (interval) and univariate ANOVA (analysis of variance, Bonferroni post-hoc test) were used to analyze differences between two and more groups of tissue samples, respectively. Pearson’s correlation coefficient was applied for correlation analysis. Cut-off-values for summary variables were determined using the receiver operating characteristic (ROC) calculation and Youden Index analysis for disease-free and overall survival (DFS, OS). For survival analysis, cases with missing date of death were excluded. Univariate survival analysis was performed using the Kaplan-Meier method comparing the survival curves with the log-rank test. For multivariate analysis, the time-dependent Cox proportional hazards model was performed to identify independent predictors for DFS and OS using the forward elimination Wald method. For all calculations, *p* < 0.05 and *p* < 0.01 were considered as significant or highly significant, respectively.

## 5. Conclusions

Taken together, our study shows that two members of the HDAC family are linked with miRNA expression in pNETs. The most interesting association is between miRNA449a and HDAC3 and HDAC4. According to our data regarding clinico-pathological, proliferative, mitotic characteristics and the survival analysis, it seems that in pNETs miRNA449a does have an oncogenic function as opposed to in other tumor entities.

## Figures and Tables

**Table 1:** Patient characteristics of pancreatic neuroendocrine tumors (pNET).

Parameters	Cases	*p* Value ^a^
Gender, *n* (%)		
Male	32 (56.1%)	0.354
Female	25 (43.9%)	
Age (mean ± SD)	60.9 ± 14.4	n.a.
Size (mean (cm) ± SD)	2.69 ± 2.34	n.a.
Localization, *n* (%) ^b^		
Head	25 (44.6)	0.067
Corpus	11 (19.6)	
Cauda	20 (35.7)	
TNM, *n* ^c^		
T1/2/3/4	24/13/17/3	** 0.001
N0/1	38/19	* 0.012
M0/1	46/11	** 0.000
Grading, *n* (%)		
G1	32 (56.1)	n.a.
G2	17 (29.8)	
G3	8 (14.0)	
R status, *n* (%)		
R0	51 (89.5)	** 0.000
R1	5 (8.8)	
R2	1 (1.8)	
OS, *n* (%)		
Yes	47 (82.5)	** 0.000
No	10 (17.5)	
DFS, *n* (%)		
Yes	34 (73.9)	** 0.001
No	12 (26.1)	
Proliferation/Mitosis		
H&E ^d^	12.8 ± 27.1	n.a.
pHH3 ^e^	15.0 ± 31.4	
Ki-67 (%) ^f^	10.8 ± 21.3	
Hormone activity *n* (%) ^g^		
No	30 (52.6)	0.691
Yes	27 (47.4)	

^a^ χ^2^ test; ^b^ Reduced case numbers due to unknown data; ^c^ according to current TNM (7th edition (2010)); ^d^ H&E-associated mitotic activity per 10 high power fields; ^e^ pHH3-associated mitotic activity per 10 high power fields; ^f^ Ki-67-associated proliferation index (% per 2000 tumor cells); ^g^ measured by immunohistochemistry; n.a., not applicable; SD, standard deviation.

**Table 2:** Cut-off-values based on overall- and disease-free-survival.

miRNA-	Mean ± SD	Cut-Off-Value (ROC, Youden-Index)	
		Overall-Survival	Disease-Free-Survival
132-3p	0.18826 ± 0.41903	0.26125	0.06320
145-5p	1.0057 ± 1.14076	0.58855	0.39820
183-5p	0.06794 ± 0.18228	0.05250	0.05250
34a-5p	0.24186 ± 0.24647	0.08215	0.29910
449a	0.00207 ± 0.00600	0.00310	0.00265

## Supplementary Materials

Supplementary materials can be found at http://www.mdpi.com/1422-0067/19/9/2781/s1

## Abbreviations

Cyt	Cytoplasmatic
DFS	Disease-free survival
EMT	Epithelial to mesenchymal transition
FFPE	Formalin-fixed paraffin-embedded
HDAC	Histone deacetylases
HPF	High power fields
IHC	Immunohistochemistry
miRNA	microRNA
nuc/ncl	Nuclear
OS	Overall survival
pNET	Pancreatic neuroendocrine tumor
SD	Standard deviation
TMA	Tissue microarray
