# Supplementary material for: HDAC-Linked “Proliferative” miRNA Expression Pattern in Pancreatic Neuroendocrine Tumors
Source: Int J Mol Sci. 2018 Sep 15;19(9):2781. doi: 10.3390/ijms19092781 (PMC6164864; doi:10.3390/ijms19092781)
Supplement: Supplementary file 1 [file ijms-19-02781-s001.pdf]

Supplementary Table S1. Antibodies and conditions used for IHC.

Antibody*	Cat.-No.	Clone / Lot No.	Dilution / incubation
HDAC1	ab19845[a]	GR100389-1	1:2000 / ½ h
HDAC2	ab16032[a]	GR114290-1	1:250 / ½ h
HDAC3	sc-11417[b]	H-99	1:100 / ½ h
HDAC4	sc-11418[b]	H-92	1:100 / ½ h
HDAC5	ab55403[a]	GR36969-3	1:500 / ½ h
HDAC6	sc-11420[b]	H-300	1:500 / ½ h
HDAC8	ab39664[a]	GR38788-6	1:100 / ½ h
HDAC9	ab59718[a]	GR129331-1	1:500 / ½ h
HDAC10	ab53096[a]	GR45168-3	1:200 / ½ h
HDAC11	ab18973[a]	GR107170-1	1:50 / ½ h
Sirt1	sc-15404[b]	H-300	1:100 / ½ h

* Species: rabbit; Pretreatment: heat-induced epitope retrieval in pH = 9 antigen retrieval buffer (Dako, Glostrup, Denmark) and waterbath; Purchased from [a] abcam (Cambridge, UK) [b] Santa Cruz Biotechnology (Dallas, Texas)

Supplementary Table S2. Correlation Analysis of miRNA and HDAC expression.

		HDAC1_PNET_ncl_Score	HDAC2_PNET_ncl_Score	HDAC3_PNET_ncl_Score	HDAC4_PNET_cyt_Score	HDAC4_PNET_ncl_Score	HDAC5_PNET_cyt_Score	HDAC5_PNET_ncl_Score	HDAC6_PNET_cyt_Score	HDAC8_PNET_cyt_Score	HDAC8_PNET_ncl_Score	HDAC9_PNET_ncl_Score	HDAC10_PNET_cyt_Score	HDAC10_PNET_ncl_Score	HDAC11_PNET_ncl_Score	SIRT1_PNET_ncl_Score
miR132_3p	Pearson's correlation coefficient	-0,194	-0,219	-0,078	-0,150	-0,196	0,006	-0,071	-0,152	-0,096	-0,054	-0,013	-0,081	-0,054	0,024	-0,115
	Sig. (two-sided)	0,181	0,127	0,600	0,314	0,186	0,968	0,633	0,292	0,523	0,703	0,929	0,591	0,722	0,871	0,435
miR145_5p	Pearson's correlation coefficient	0,256	0,126	0,033	-0,046	0,047	-0,003	0,056	-0,062	0,033	0,004	0,019	0,088	0,062	-0,041	-0,091
	Sig. (two-sided)	0,076	0,384	0,826	0,760	0,752	0,981	0,704	0,670	0,826	0,979	0,893	0,559	0,681	0,783	0,536
miR183_5p	Pearson's correlation coefficient	-0,113	-0,092	-0,153	-0,196	-0,208	-0,102	-0,072	-0,195	-0,008	-0,036	-0,091	0,198	0,143	-0,129	-0,206
	Sig. (two-sided)	0,438	0,526	0,298	0,187	0,160	0,491	0,627	0,174	0,958	0,802	0,522	0,186	0,341	0,383	0,160
miR34a_5p	Pearson's correlation coefficient	-0,125	-0,086	0,194	0,012	0,152	-0,002	0,010	0,006	0,152	-0,003	0,088	0,083	0,060	-0,038	-0,003
	Sig. (two-sided)	0,393	0,554	0,186	0,936	0,308	0,987	0,947	0,966	0,309	0,985	0,535	0,582	0,692	0,796	0,982
miR449a	Pearson's correlation coefficient	0,237	0,192	0,310	0,302	0,245	-0,179	0,154	0,042	0,045	0,001	-0,008	-0,042	-0,099	0,107	0,176
	Sig. (two-sided)	0,101	0,182	**0,032**	**0,039**	0,097	0,224	0,294	0,771	0,765	0,995	0,955	0,782	0,513	0,469	0,231

Pearson correlation analysis of miRNA expression data and HDAC expression data. Shown are the correlation coefficients and the p-values. * $p < 0.05$, ** $p < 0.01$.

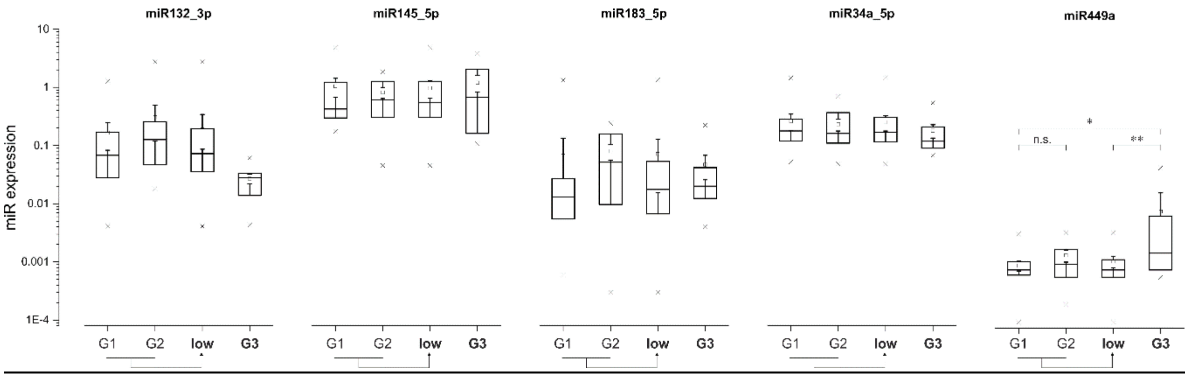

Supplementary Figure S1. Quantitative analysis of pNET-miRNA expression versus grading. The semi-quantitative miRNA expression scores are plotted via box plots versus grading of pNET cases. Additionally, the expression levels were summarized for G1 and G2 pNET cases. * p<0.05, ** p<0.01 (t-Test and ANOVA). According to statistical analysis there are only significant group differences related grading and miRNA449a.

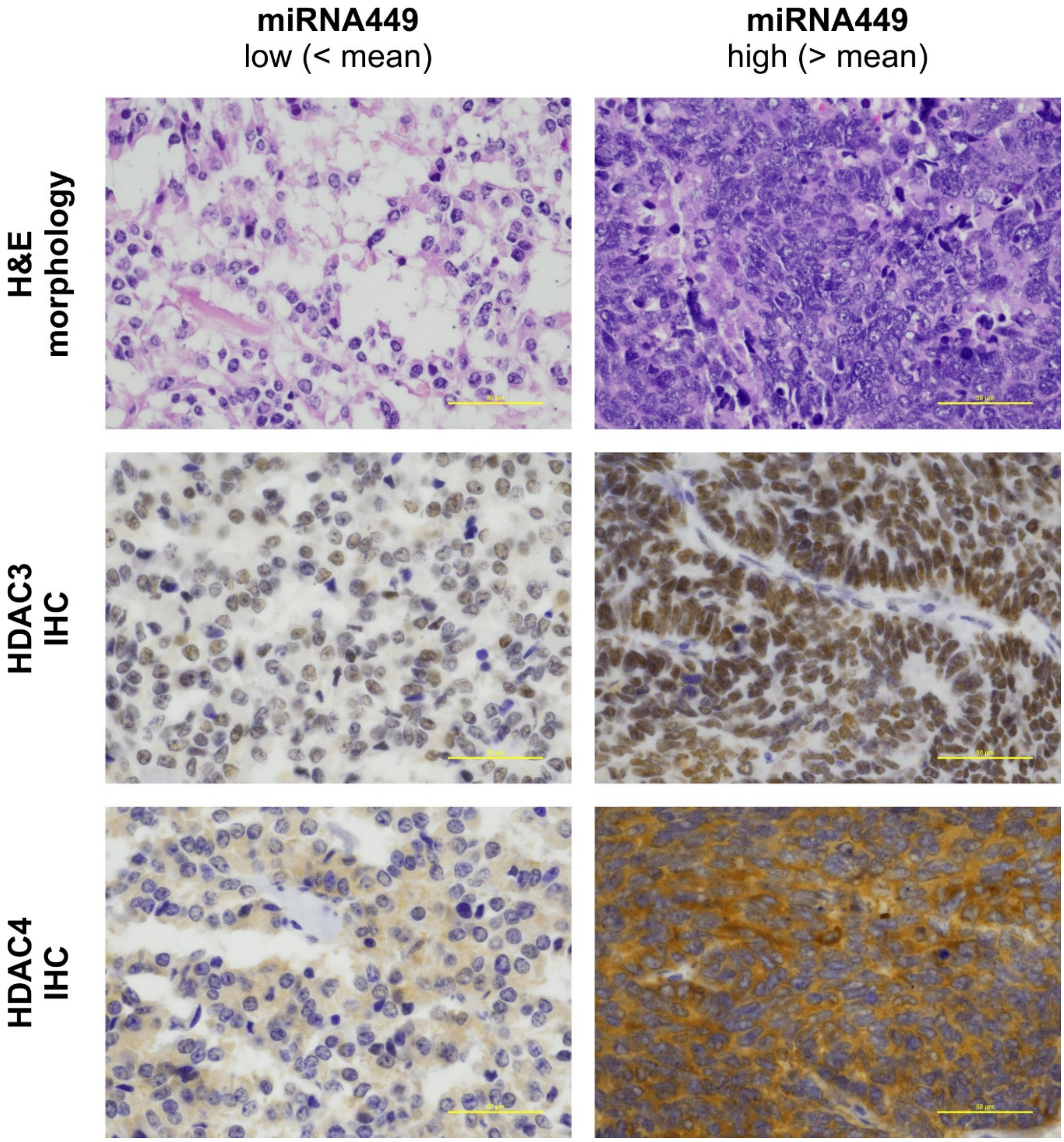

Supplementary Figure S2. Histomorphology and HDAC3/4 Immunohistochemical staining. Representative images of H&E morphology (top row) and HDAC3 (second row) and HDAC4 (third row) immunohistochemistry staining for pNET cases with significant differences in HDAC expression related to miRNA449a expression levels (low/high). Scale bar (yellow) indicates 50µm at 400x magnification. Abbreviations: HDAC, histone deacetylase; H&E, hematoxylin & eosin; IHC, immunohistochemistry; pNET, pancreatic neuroendorcine tumor.
